# Effects of Distance, Noise, and Personal Respiratory Protective Equipment on Speech Comprehension in Simulated Critical Care Setting

**DOI:** 10.3390/healthcare13040398

**Published:** 2025-02-12

**Authors:** Andrés Rojo-Rojo, José Eugenio Martínez-Abril, Gloria María Muñoz-Rubio, Paloma Iniesta-Cortés, Juan Manuel Cánovas-Pallarés

**Affiliations:** 1 Intensive Care Unit, Virgen de la Arrixaca Universitary Hospital, Public Murcian Health Service, 30120 Murcia, Spain; 2 Faculty of Nursing, Catholic University of Murcia (UCAM), 30107 Murcia, Spain; 3 Emergency Healthcare Sistem, Public Valencian Health Service, 03010 Alicante, Spain

**Keywords:** speech intelligibility, personal protective equipment (PPE), environmental noise, critical care, word comprehension

## Abstract

**Background/Objectives:** Effective communication in critical care is crucial, particularly with the constraints of Personal Protective Equipment (PPE). This study aimed to evaluate speech intelligibility under varying conditions of distance, ambient noise, and PPE types in a simulated ICU. **Methodology:** A quasi-experimental design was used with 23 participants in 24 scenarios, combining three distances (1, 2, and 5 m), two noise levels (quiet and ICU-like), and four PPE types (no mask, surgical mask, N95, and PAPR). Speech intelligibility was assessed by positioning the subjects at varying distances while playing phonetically balanced words through a playback device equipped with the different PPE being tested. The evaluation was conducted under two sound conditions (25 dB(A) and 45 dB(A)). The percentage of correct responses by the subjects to the perceived sounds was determined as a measure of intelligibility. The relation between variables was analyzed using the Wilcoxon Test and the Friedman Test. **Results:** Significant differences in word recognition were observed across conditions. Ambient noise reduced intelligibility, with recognition percentages significantly lowering in noisy environments. PPE type also influenced comprehension, with PAPR posing the greatest challenge. Friedman’s test showed that increasing distance significantly decreased recognition accuracy. Distances beyond two meters negatively impacted intelligibility across all devices tested. **Conclusions:** In noisy conditions (>45 dB(A)), a combination of distances greater than two meters and PPE use reduced intelligibility by over 40%, potentially compromising patient safety. The use of communication aids, such as visual cues or alternative speech devices, is recommended, particularly with PAPRs, to enhance comprehension and ensure effective communication between healthcare providers and patients.

## 1. Introduction

Face masks or face shields are routinely used in healthcare settings by medical staff to prevent both contact and respiratory exposure to patient secretions, droplets, and airborne particles. The use of face masks significantly reduces the transmission of respiratory droplets that may carry hazardous viruses, a critical measure particularly emphasized during the COVID-19 pandemic [1].

In clinical settings, especially since the COVID-19 outbreak, and previously to protect against other respiratory-transmitted infections, healthcare workers have relied on face masks as a key protective measure. These masks serve to prevent the spread of airborne particles and droplets, thereby reducing the risk of transmission to infected patients. In particular, the use of masks has been instrumental in curbing virus transmission during this period [1,2].

However, despite these protective benefits, the use of respiratory protective masks is not without drawbacks. Common issues include discomfort, difficulty breathing, headaches, and, perhaps most critically, impaired oral communication [3,4,5]. The negative impact on the speaker’s ability to communicate effectively and the listener’s capacity to comprehend speech presents a significant challenge [6]. These discomforts often result in diminished compliance with mask-wearing protocols, as individuals tend to frequently touch, adjust, or remove their masks, increasing the risk of exposure during these moments [7].

Clear and effective communication is indispensable in nursing care, particularly in emergency scenarios and when managing critically ill patients [8,9]. In these high-stakes environments, it is essential that all parties—healthcare professionals as well as non-professional staff—engage in clear dialog, ensuring that messages are conveyed and understood without ambiguity. Emerging evidence highlights that the use of Personal Protective Equipment (PPE), including face masks, significantly impairs speech intelligibility, which may hinder effective communication [10,11,12,13,14].

Moreover, studies indicate that the use of PPE, especially face masks, adversely affects nurses’ ability to communicate effectively within hospital settings, potentially contributing to serious medical errors [15]. Additionally, a key factor influencing speech intelligibility is the signal-to-background noise ratio, as high levels of background noise can severely impair communication among healthcare professionals, further increasing the likelihood of accidents due to a combination of miscommunication and reduced concentration [16].

Intensive Care Units (ICUs) are notoriously noisy environments, even under relatively calm conditions. Research by Darbyshire and Young [17] has shown that baseline noise levels in five different ICUs did not fall below 45 dB(A), with peak levels reaching up to 90 dB(A) during the day and 70 dB(A) at night. These sources of noise include conversations among staff, equipment alarms, electronic devices, and the movement or handling of objects [18].

The World Health Organization (WHO) has established recommended noise exposure limits for hospitals: up to 40 decibels per hour during daytime shifts and 35 decibels per hour during night-time [19]. However, there is limited research on how masks impact voice transmission under varying signal-to-noise ratio conditions. Studies such as that by Corey et al. [20] have demonstrated that different types of masks possess distinct acoustic properties. Randazzo et al. [21] indicated that although all masks reduce speech signal clarity, N95 masks have the most pronounced effect. Consequently, individuals wearing these masks often raise their voice to compensate, which can strain the vocal cords and lead to issues such as dysphonia and other voice disorders [22,23,24].

In today’s clinical environment, verbal communication is not only hindered by background noise but also by the widespread use of respiratory protective devices like face masks. This combination can significantly affect how listeners perceive and interpret messages from the speaker. Wittum [25] demonstrated that the percentage of correct sentence recognition was high without a mask, but dropped to 33.1% with a mask and further to 20.9% when both a mask and face shield were used.

Previous research has extensively documented the effects of respiratory protective masks on speech intelligibility [26,27], establishing that such devices interfere with the transmission of verbal messages. Building on this foundation, our study aims to investigate the impact of different respiratory protection systems—surgical masks, KN95, and a Powered Air Purifying Respirator (PAPR)—on speech discrimination and the distance at which speech intelligibility is compromised in the context of caring for critically ill patients. Additionally, we examine how ambient noise further exacerbates these challenges, potentially hindering effective communication among healthcare professionals. We hypothesize that the use of these respiratory protection systems, combined with high levels of background noise, significantly reduces speech discrimination and limits the distance at which verbal communication remains intelligible in such high-stakes environments.

## 2. Materials and Methods

### 2.1. Study Design

This is a quasi-experimental study conducted between January and February of 2024. The steps outlined in the TREND statement (Transparent Reporting of Evaluations with Non-Randomized Designs) were followed in conducting this study. The study employed a design comprising 2 (Ambient Noise: 25 dB(A) vs. 45 dB(A)) × 3 (Distances: 1 m, 2 m, 5 m) × 4 (Personal Protective Equipment: None, Surgical Mask, FFP2 Mask, PAPR) conditions.

### 2.2. Study Population and Sampling Strategy

Two groups of students were randomly selected from among the 25 groups engaged in clinical simulation activities within the Nursing degree program at the Catholic University of Murcia UCAM, with an age range of 19 to 40 years, and a 70–30 ratio of women compared to men. A sample of 23 subjects was formed. No sample size calculation was performed. The decision not to calculate the sample size was primarily driven by practical considerations related to the specific context of the research. Similar surveys included samples of a similar or smaller size [26,28,29].

The inclusion criteria for participation in the study included having normal hearing (i.e., no hearing impairments such as any degree of deafness), students studying clinical simulation, and students familiar with the clinical simulation rooms at UCAM. All participants were informed that the aim of this research was to assess speech perception when using different PPE. After being informed about the study, each participant completed a consent form. The exclusion criteria for selecting participants included the presence of voice disorders, respiratory conditions, or infections (such as colds or flu), or disorders of the auditory tract (such as otitis).

### 2.3. Devices and Experimental Context

**Figure 1:**
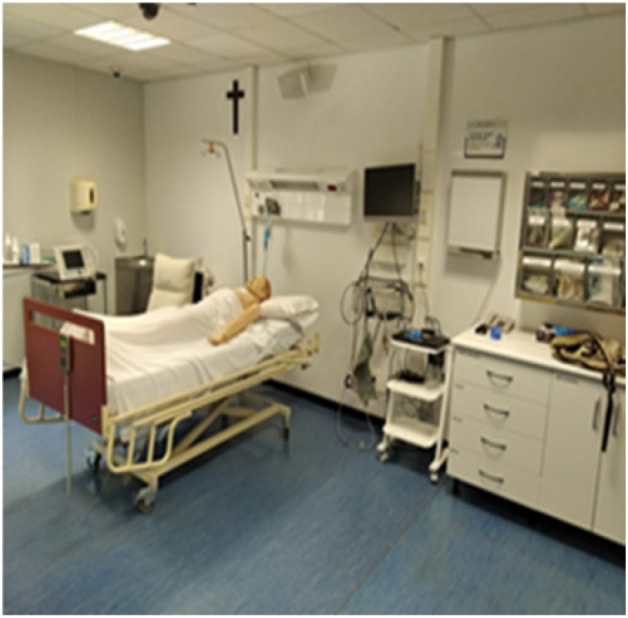
UCAM Clinical Simulation Room.

The experimental process was conducted in a high-fidelity simulation room at UCAM, designed for training in nursing and medicine. This room (Figure 1) replicates a real clinical environment, similar to an emergency or intensive care unit, equipped with the inventory and materials used in such settings. This setup creates the characteristic reverberation and acoustic opposition found in these environments. Ambient noise was played in the room using background sounds recorded from an actual intensive care unit at a volume of 45 dB(A), which is comparable to the routine background noise in an ICU, as supported by the available evidence.

**Figure 2:**
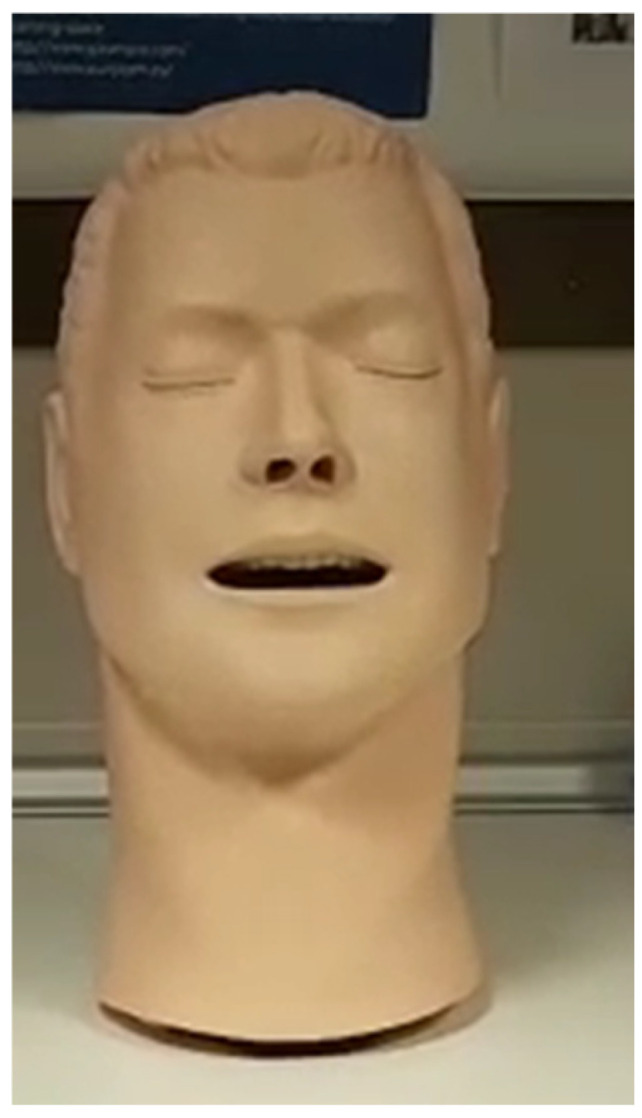
Laerdal Airway Management Trainer©, UCAM property.

For sound emission, an anatomical head model from a Laerdal Airway Management Trainer© (Laerdal Medical, Madrid, Spain) (Figure 2), commonly used for oro-tracheal intubation training, was utilized. Inside the vocal cavity, a Bluetooth speaker (Sony SRS-XB13© Sony, Madrid, España, small size—width: 76 mm; depth: 76 mm; height: 95 mm, with a sound power of 5 W and a frequency range of 20 to 20.000 Hz) was sealed within the mouth using sound-absorbing polyurethane foam to minimize internal sound reverberation. An opening measuring 6 × 30 mm at the lip allowed the head to emit voice signals and noise. The different respiratory protection devices (face masks and PAPR equipment) were mounted on or over the head.

This device was positioned at a height of 1.70 m from the floor (the average height of a healthcare professional), maintaining a distance of at least 1 m from the walls and controlled distances of 1, 2, and 5 m from the listener.

For sound measurement, a sound level meter, the Trotec Soundmeter SL400©, Trotec, Barcelona, Spain, was used. This is class II equipment, a device commonly used in the documentation of noise produced by machines or in work environments. It complies with European standards for recording noise in the working environment. It provides dB(A) and dB(A) readings. The dB(A) was used as the reference sound measurement for this study. dB(A) is the acronym for “A-weighted adjusted decibels”, a magnitude that eliminates the highest and lowest frequencies of sound, which are imperceptible to the human ear. The main advantage of this reference compared to others is that it more accurately reflects how the human ear perceives sound in terms of intensity and annoyance. For this reason, it is commonly used as a reference in environmental and occupational noise assessments.

To measure the baseline characteristics of the sound emitted by the speaker, a reference sound level meter was placed next to the mannequin’s head. Given that, in a normal conversation between two people in a quiet environment, voice intensity may range from approximately 45 to 55 decibels [28], sounds were emitted within these decibel ranges; the emitted voice had an intensity of 55 dB(A), measured at a distance of 1 m from the speaker’s exit, at the same height.

To represent the ambient sound of an ICU, background sounds recorded from an actual ICU were played through the room’s speaker system. This ambient sound recording was looped during the testing process. The sound devices in the simulation rooms were integrated into the corners of the space, allowing the sound to converge in the center of the room where the assessments took place. The ambient sounds were emitted at an intensity of 45 dB(A), which is below the recognized sound levels in various ICUs.

### 2.4. Respiratory Protection Equipment: Respiratory Masks and PAPR Equipment

In this study, three different types of PPE were utilized: a surgical mask, an N95 mask, and a 3M Powered Air-Purifying Respirator (PAPR) face shield, alongside a no-mask condition to simulate real-world conversation scenarios.

The surgical mask consists of three layers. The innermost layer, which is in direct contact with the face, is typically made of nonwoven polypropylene fabric (TNT) or cellulose. According to Spanish UNE standards, this layer must be composed of 100% spunbond polypropylene with a weight of 20 g/m^2^ [29]. Surgical masks are designed primarily for protection against large droplets and splashes, making them suitable for various healthcare settings.

In contrast, KN95 masks, also referred to as FFP2 masks in Europe, are constructed with four or five layers, with three filter layers in the middle. These internal filter layers are typically made from a thin, densely bonded layer of spun-bond polypropylene fibers. The density of these layers is crucial for their filtration efficiency, as they capture smaller particles, including aerosols, thus providing a higher level of protection compared to standard surgical masks.

**Figure 3:**
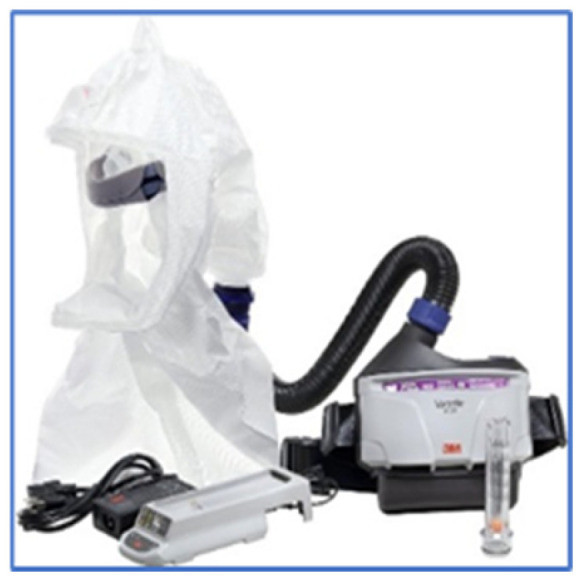
3M Versaflo© TR-300MR blower, UCAM property.

The PAPR (Powered Air-Purifying Respirator) (Figure 3) system features a hood that covers the wearer’s head and is connected to a ventilation unit that supplies filtered air from the external environment. This system is particularly advantageous in high-risk situations, as it provides a continuous flow of clean air. In this study, the equipment used included the 3M Versaflo© TR-300MR blower, 3M Madrid, Spain, (weighing 1095 g), which is worn around the waist with a belt, accompanied by a head harness and a hood extending to the shoulders (model S-655-L). This ventilator maintains a nominal airflow rate of 190 L/min, ensuring adequate respiratory protection while enhancing comfort during prolonged use, making it a valuable option for healthcare professionals working in environments with a high risk of airborne pathogens.

### 2.5. Experimental Design I: Conditions of the Experiment and Manipulation of Variables

Three variables were manipulated: the distance between the emitter and the receiver, the ambient noise in the room, and the type of protective equipment worn by the emitter.

For the distance between the emitter and receiver, distances were marked on the floor of the room at 1, 2, and 5 m. The emitter was placed on a stable tripod at a height of 1.70 m, simulating the standard height of a healthcare professional. Between the emitter (head with speaker) and the receiver (subject of study), a bed with a patient was positioned, surrounded by everyday objects typically found in an ICU (monitors, ventilators, intravenous infusion pumps) to generate acoustic effects similar to those encountered in routine practice. These distances were chosen to represent the following: the first distance represents the standard distance between two people on either side of a patient’s bed; the second represents the internationally established interpersonal safety distance; and the third indicates the maximum distance between a person attending to a patient while using respiratory protection equipment and the outside of the room.

For the ambient noise variable, two distinct situations were considered: without ambient noise and with ambient noise similar to that of an ICU. In the first case, identified as “Sound Condition 0: Without Ambient Noise,” the acoustic conditions of the room were maintained in a natural state. A mean sound level of 25 dB(A) was recorded in silence within the room. This condition is comparable to “silence”. The second condition, identified as “Sound Condition 1: ICU Ambient Noise,” involved the playback of pre-recorded sounds of ICU environments through stereo speakers integrated into the room, positioned in each corner. These sounds were played at a sufficient volume to register an average sound level of 45 dB(A) in the center of the room, similar to that found in an actual ICU during moderate activity. A diagram of the experimental procedure followed can be found in Figure S1 of the Supplementary Materials.

For the type of protective equipment worn by the emitter, various types of protective equipment were placed on the emitter’s head: a surgical mask, an NK-95-FFP2 mask, and a PAPR device. Additionally, measurements were taken in the absence of protective equipment on the emitter. This resulted in the four previously mentioned conditions: without a mask, with a surgical mask, with an NK95-FFP2 mask, and with a PAPR device.

Therefore, the combination of the various configurations of these three variables yielded a total of 24 possible situations for each receiving subject.

### 2.6. Experimental Design II: Word Recognition and Sound Emission Conditions

Different sequences of words were reproduced through the speaker located in the mouth of the mannequin, selected from a list of phonetically balanced words. A total of 12 phonetically balanced word sequences were composed for the Spanish language (non-Latin). The words chosen for each sequence were extracted from the PIP-C50 recording sheet [30]. The 12 selected word sequences can be found in Table S1 of the Supplementary Materials.

To minimize the interference from the microphone that reproduces sound via the speaker, the different word lists were pre-recorded using conventional recording software. All sequences were recorded in an anechoic chamber belonging to the audiovisual resources department of UCAM. An interval of 2 s was maintained between each word. A female voice was used for the narration, ensuring a consistent speaking level (tone, intonation, and timbre) throughout each voice recording. The emitted message was delivered in a neutral tone, without emphasizing any words, and at a normal speaking rate and normal level of clarity.

The emitted signal was modulated and equalized, producing sound within a frequency range of 100 Hz to 10 kHz at a level of 55 dB(A) one meter from the mouth reference point, akin to what would be emitted during a normal conversation.

To achieve this, the researcher in the room randomly selected a number between 1 and 12. This number corresponded to a list of pre-recorded words, which were then played in the experimental conditions being tested at that moment. This process was repeated twice to establish 24 experimental scenarios. Thus, each subject was only exposed to the same sequence twice during the test, minimizing the impact of memory bias.

### 2.7. Experimental Design III: Experiment Protocol

All study participants were welcomed and asked to wait in a room adjacent to the experimental area. In this room, the research team (A.R.R., G.M.M.R., P.I.C.) reviewed the experimental conditions, verified the inclusion criteria, explained the informed consent process, and reiterated the participants’ right to withdraw from the study at any time. The entire process was estimated to take approximately 15 min.

For this purpose, four continuous rooms were available: room A (waiting room), room B (control room), room C (simulation room), and room D (debriefing room).

Participants were also informed that the aim of the study was to assess the perception of speech under different PPE conditions. The order of entry was determined based on the convenience of the participants, taking into account their waiting times and personal schedules.

A designated member of the research team (P.I.C.) was responsible for welcoming participants, managing their entry and exit, providing instructions, offering feedback, and handling goodbyes.

Inside room C, another researcher (A.R.R.) guided the participants, explained the required positions, replaced the manikin’s PPE, and recorded participants’ verbal responses to the manikin’s speech. Participants were explicitly informed about this process and instructed to repeat aloud the words they perceived, without interpreting or modifying them. Any uncertainty in their responses was considered an error in comprehension. The researcher in the experimental room refrained from answering, as this might influence the participants’ responses.

In room B, a third researcher (G.M.M.R.) was in charge of selecting and randomizing the word lists presented to each participant. This researcher controlled the playback of the words through the dummy speaker and monitored the sound conditions in the experimental room, alternating between Sound Condition 1 and Sound Condition 0 as needed.

### 2.8. Data Collection Tools

The speech intelligibility test was performed using the speech discrimination scoring. Speech discrimination is the ability to understand speech in both quiet and noisy environments. Although speech can be detected if it reaches a sufficient volume, understanding it can be difficult for some people, especially in the presence of noise [31].

To facilitate this task, a researcher in the room recorded whether the uttered word was understood correctly. The researcher in the room was unaware of the playlist that would be broadcast; he behaved blindly in this task. His task was to record the word repeated aloud by the research subject and orient the subject to the different positions to be occupied in the room.

The intelligibility margins were established based on the IEC 60268-16 standard recommendation [32] and on similar previous studies [33,34].

Participants verbally pronounced the word they understood and the researcher in the simulation room documented these responses in writing on a recording sheet. This sheet included information on the participant’s characteristics (gender, age, and personal identification number), the type of PPE worn by the head-speaker device, the distance, and the presence of ambient noise. The control room researcher also recorded the list of words selected for each of the experimental conditions for each subject. At the end of the test, the control room researcher and the simulation room researcher combined the documents for each subject. By subsequently comparing both documents, whether the word played was the word recognized by the subject was determined, thus obtaining the results of the intelligibility test.

At the end of the experiment, the percentage of concordance between the answers provided and the message transmitted was calculated, obtained using the number of hits for the words recognized by the listeners from the original message. This “percentage of intelligibility” was added up for each individual, providing an average for each of the conditions. This average percentage of agreement was classified on the basis of the following criteria: Excellent (100–91%)—only the highest scores were rated as outstanding; Good (90–81%)—reflects solid performance without reaching excellence; Regular (80–61%)—expands the mid-range category, potentially indicating areas for improvement; Poor (60–41%)—identifies minimum tolerable limits; Bad (40–21%)—highlights obvious problems requiring attention; Very Bad (<21%)—indicates unacceptable performance.

This classification allowed for a stricter assessment of performance by better distinguishing the higher levels (Excellent, Good) and highlighting critical areas (Poor, Very Poor). This approach is useful when the analysis is intended to identify areas for improvement or to set high performance standards.

### 2.9. Statistical Analysis

The dataset was analyzed using Microsoft Excel and SPSS v21.0 (IBM Corporation, Armonk, NY, USA). Descriptive statistics were generated and normality was assessed using the Shapiro–Wilk test, which is well-suited for small to medium sample sizes. The test results indicated that the data did not follow a normal distribution, supporting the decision to apply non-parametric statistical methods for subsequent analyses.

The study considered three independent variables: ambient sound conditions (two levels), emitter–receiver distance (three levels), and respiratory protective equipment (four conditions). For the descriptive analysis, the percentage of correct responses, averaged and recorded at each experimental station, was used.

The Friedman test was used to evaluate the relationships between the three principal dependent variables and the independent variable. Additionally, the Wilcoxon signed-rank test was performed to compare the two ambient sound conditions in pairwise analyses.

Based on the potential results of the variance analysis tests, the following hypotheses were formulated. Hypotheses were established for each variable analyzed: (a) Impact of respiratory protective equipment (PPE): H_1_ (Alternative Hypothesis): “There are significant differences in word recognition among the various types of respiratory protective equipment”. H_0_ (Null Hypothesis): “There are no differences in word recognition among the various types of respiratory protective equipment”. (b) Impact of ambient noise: H_1_ (Alternative Hypothesis): “Ambient noise significantly influences word recognition”. H_0_ (Null Hypothesis): “Ambient noise does not influence word recognition”. (c) Impact of emitter–receiver distance: H_1_ (Alternative Hypothesis): “Distance between interlocutors significantly affects word recognition”. H_0_ (Null Hypothesis): “Distance does not affect word recognition”.

### 2.10. Ethical Considerations

Ethical approval for this study was obtained through the Ethics Committee of the UCAM, protocol report number CE122201. The participants were informed of the purposes of the study, the conditions, and the absence of risks, as well as the time that would be involved in carrying out the study.

## 3. Results

### 3.1. Participant Analysis

Data were collected from 23 subjects, all belonging to two groups of 4th year nursing students. The results are shown in Table 1. All participants met the inclusion/exclusion criteria. The participants had a median age of 22 years, with 70% being women (n = 16) and 30% men (n = 7).

To conduct a descriptive analysis of the data, we differentiated the results based on the various variables. When examining the impact of ambient noise (Table S2) on the number of correctly recognized words by the subjects, we observed a significant negative effect of ambient sound. In other words, under the most favorable condition (at one meter, with no mask on the speaker), the average word recognition in Condition 0 was 4.78 words, or 95% of all words spoken. However, under the same experimental condition (at one meter, with no mask on the speaker), the number of correctly identified words dropped to 4.13 representing a decrease of 0.65 words, or a 13% reduction in word recognition, due to the effect of ambient noise, even under the best experimental condition tested.

This effect notably worsened with increased distance and the use of different respiratory protection devices. The worst results for word comprehension were obtained in Condition 1, at a distance of 5 m, with the speaker wearing a PAPR. Under these conditions, the average number of correctly identified words was 1.21 out of 5 words spoken, meaning that less than 25% of the words were correctly understood, indicating a significant impairment in communication (Table 2).

An initial examination of the data tables suggested that the data do not follow a normal statistical distribution. Therefore, non-parametric tests were used to analyze the relationship between the variables.

### 3.2. Descriptive Analysis of Percentage of Correct Responses

Given that the data do not follow a statistically normal distribution and the Friedman and Wilcoxon tests yielded significant results, we performed a descriptive analysis of the correct responses for each experimental condition, grouped by variables. This allowed us to descriptively examine the percentage of correct responses and how it is affected by the variables.

### 3.3. Descriptive Analysis of Experimental Data

To conduct a descriptive analysis of the data, the results were differentiated based on the various variables. Specifically, the percentage of correct recognitions (intelligibility percentage) was analyzed under each of the given conditions (distance, Respiratory Protection Equipment, and ambient Sound). The data were categorized as described in Section 2.8. The number of subjects included in each category is presented, with the % intelligibility provided in parentheses.

#### 3.3.1. Impact of Distance Between Interlocutors on Message Comprehension

The effect of distance on the percentage of correct responses is presented in the attached table (Table 3).

At the 1 m mark, six out of the eight experimental conditions tested show high intelligibility percentages, predominantly above 80%. Only two experimental conditions exhibit intelligibility percentages below 80%; both occurred under ambient noise conditions (>45 dB(A)), particularly when using the PAPR.

At the 2 m mark, the number of conditions with intelligibility percentages above 80% decreases to four out of eight. In other words, the conditions with intelligibility percentages below 80% increase to four. This indicates a reduced understanding of words as the distance doubles. At two meters, Excellent results decrease by 25% compared to those at one meter, while the conditions reflecting impaired understanding double. Of these six conditions with results of below 80% intelligibility, three occurred under ambient noise conditions (>45 dB(A)), and both PAPR conditions are included in this category.

Finally, at the 5 m mark, the number of conditions with intelligibility percentages above 80% drops to three out of eight. That is, the conditions with intelligibility percentages below 80% exceed those with high recognition rates. At five meters, excellent results decrease by 50% compared to those at one meter. All conditions with percentages below 80% occurred under ambient noise, and all conditions involving the use of PAPR as respiratory protective equipment fall into this category.

By observing the percentages of correct responses, we can deduce the influence of distance on the accurate reception of messages, with five meters being particularly affected, especially in the presence of ambient noise and when occlusive masks are used. Notably, the use of a PAPR by the speaker at this distance plays a particularly negative role. Under noisy conditions, with a five-meter gap between interlocutors, nearly 50% of participants correctly understood fewer than 1 in 5 words, showing a serious impairment to verbal communication.

#### 3.3.2. Impact of Respiratory Protection Equipment on Message Comprehension

The effect of respiratory protective equipment on the percentage of correct responses is presented in the attached table (Table 4).

In the “No Mask” experimental condition, five out of the six tested conditions achieved intelligibility results above 80% in the majority of cases. The only condition with intelligibility below 80% was at a five-meter distance with ambient noise (>45 dB(A)).

When the speaker wore a surgical mask, intelligibility remained above 80% in four out of six conditions, surpassing the baseline (no mask). The two conditions with intelligibility below 80% were those with ambient noise at distances greater than 1 m (2 and 5 m).

With the FFP2 mask, intelligibility exceeded 80% in three of the six conditions. All conditions with ambient noise at the three tested distances (1, 2, and 5 m) resulted in an intelligibility below 80%.

Finally, with the last PPE tested (PAPR), only one condition out of six showed an intelligibility above 80%: no noise at a one-meter distance, the most favorable condition. In all other conditions, intelligibility was predominantly below 80%. This device performed the worst of all, showing intelligibility below 20% in 30% of subjects, a result seen exclusively in the worst test condition (five meters with ambient noise).

The data shown for the experimental condition of the speaker not wearing a mask suggest that noise and increased distance can significantly hinder communication, even without any protective equipment.

With these data, we observed that the presence of a surgical mask had a relatively minimal impact on intelligibility, especially in noise-free conditions. However, in ambient noise conditions at distances greater than 1 m, intelligibility decreased, highlighting the limitations of surgical masks in noisy environments.

The FFP2 mask, while offering increased filtration, may compromise communication in noisy or distant interactions, potentially limiting its effectiveness in real-world environments, such as clinical or industrial settings, where both noise and distance are commonplace.

These data raises questions about the feasibility of using PAPR devices in environments where clear communication is essential, as they can significantly affect the understanding of speech, especially in challenging conditions such as those involving distance or background noise. The device performance, with intelligibility below 20% in 30% of subjects in the worst conditions, suggests the need for further improvements in PAPR technology to mitigate its negative impact on speech communication.

#### 3.3.3. Impact of Ambient Noise Conditions on Message Comprehension

The effect of ambient noise on the percentage of correct responses is presented in the attached table (Table 5).

Of the 12 experimental conditions with ambient noise levels around 25 dB(A), 10 show intelligibility percentages above 80% for the majority of the cases. The two conditions where comprehension is 80% or lower involve the use of PAPR respiratory protection and distances greater than one meter (2 and 5 m).

This suggests that, under low ambient noise conditions, experimental subjects can recognize at least four out of every five spoken words in most experimental conditions, regardless of distance or whether the speaker wears a surgical mask or an FFP2 mask. In contrast, in conditions with noise levels exceeding 45 dB(A), only three of the twelve tested conditions show an intelligibility above 80% for the majority of cases. These three conditions are at 1 and 2 m without a mask, and at 1 m with a surgical mask. All other experimental conditions show intelligibility percentages below 80% for the majority of the cases.

The worst intelligibility results were obtained in experimental conditions with noise at a distance of 5 m, regardless of the type of respiratory protection used. In these conditions, the majority intelligibility percentage was 60% or lower, with the PAPR condition at 5 m in ambient noise standing out negatively. In this condition, intelligibility was below 20% in more than 30% of the subjects.

These results provide critical insights into the impact of ambient noise and respiratory protection on intelligibility. Under low ambient noise conditions (around 25 dB(A)), the majority of conditions resulted in intelligibility rates above 80%, suggesting that, in quieter environments, the transmission of speech is generally not hindered by the type of respiratory protection used. This is particularly notable as even the use of surgical or FFP2 masks did not significantly affect intelligibility at close distances. However, as the distance between the speaker and listener increased, intelligibility was compromised, especially when using PAPR protection, indicating that while masks offer protection, they might still present communication challenges in certain settings.

In contrast, when ambient noise levels exceeded 45 dB(A), intelligibility rates dropped significantly, with only a few conditions maintaining an intelligibility of above 80%. This highlights the detrimental effect of background noise on speech recognition, even in more favorable conditions such as shorter distances and the use of a surgical mask. The particularly poor performance of PAPR protection, especially at a 5 m distance with ambient noise, raises serious concerns about its effectiveness in environments where clear communication is critical. The fact that intelligibility dropped below 20% in over 30% of subjects in the most challenging conditions suggests that PAPR may not be suitable for situations requiring high levels of verbal communication, such as healthcare or emergency settings, without additional adjustments.

Overall, these findings emphasize the need for careful consideration when choosing the means of respiratory protection in environments with high noise levels or at greater distances. It is clear that while respiratory protection is essential, its impact on communication needs to be accounted for, particularly in situations where clear, efficient verbal interaction is critical.

#### 3.3.4. Relational Analysis Among Variables

The descriptive analysis of the results revealed a relationship among the variables of ambient sound, the use of respiratory protective equipment, and distance in the interpretation of emitted words. The analysis was conducted using data from 23 subjects, each exposed to the different experimental conditions, focusing on the total number of correct responses rather than the intelligibility percentage, as was conducted in the descriptive section. After analyzing the characteristics of the population and identifying the data as statistically non-normal, non-parametric tests were applied to analyze the relationships among the variables. Specifically, the Friedman Chi-Square test and the Wilcoxon test were employed to examine the associations between the independent and dependent variables.

To explore these relationships—ambient sound conditions, distance between interlocutors, and types of respiratory protective equipment—and the dependent variable, word recognition accuracy, a bivariate analysis was conducted. This approach facilitated the identification of potential associations and the assessment of statistical significance for each variable’s influence on the ability to correctly recognize spoken words. The analysis utilized non-parametric methods to account for the non-normal distribution of the data and the repeated-measures design of the study. Statistical significance was determined using the *p*-value, with a threshold of 0.05, to assess whether the observed relationships were meaningful. The results provide valuable insights into how environmental factors and respiratory protection interact to impact speech intelligibility under controlled experimental conditions.

##### Friedman Chi-Square Test

A bivariate analysis comparing each dependent variable to the independent variables using the Friedman test are shown in Table S3.

Regarding the relationship between the use of PPE and word recognition, the *p*-value is much lower than 0.05 (*p* = 1 × 10^−6^), allowing us to reject the null hypothesis, which posits that there are no differences in word recognition among the different masks. In other words, a significant difference in word recognition exists among the various types of masks. This indicates that the different types of respiratory protective equipment tested significantly affect word recognition. The characteristics of the respiratory equipment used influenced intelligibility, or the number of words that subjects could comprehend, in this experiment.

A similar effect was observed regarding the impact of ambient noise on word recognition in this context. It can be inferred that there is a statistically significant influence between ambient noise and the number of recognized words; in other words, in this type of environment, more ambient noise leads to poorer understanding.

Regarding the influence between distance and word recognition, this relationship is not statistically significant, as the significance value is greater than 0.05, (*p* = 0.171. Therefore, a theoretical independence between the studied variables should be assumed. In other words, word recognition was not affected by the distance at which the word was recorded under the studied conditions.

##### Wilcoxon Test

For a more detailed analysis of the impact of the different conditions of the studied variables, the Wilcoxon test was applied to specific conditions for each variable. In the analysis of the influence of distance on word recognition by listeners, two extremes were considered, 1 m and 5 m, representing the potential range of effect. Regarding the influence of ambient sound on recognition, a comparison was made between the conditions with no ambient sound (<25 dB(A)) and with added ambient sound (>45 dB(A)) to evaluate its specific impact. Finally, for the analysis of the impact of different PPE devices, the effect of each tested PPE (surgical mask, FFP2 mask, and PAPR) was assessed against the condition without any mask, which served as the null condition for comparison.

Thus, the Wilcoxon test was conducted across five conditions to analyze the following relationships: (a) without noise vs. with noise; (b) distance 5M vs. 1M; (c) PAPR vs. no mask; (d) FFP2 vs. no mask; and (e) surgical mask vs. no mask. This approach allowed for a focused comparison of the effects of each variable on word recognition accuracy. The results of the Wilcoxon test are presented in Table S4.

Based on these results, for the relationship between without noise vs. with noise (ambient sound vs. no ambient sound), since the Z value is −4.202 and the asymptotic significance (*p*-value) is 0.0000, and because the *p*-value is well below the 0.05 threshold, the null hypothesis can be rejected. This indicates a statistically significant difference in word recognition between conditions with and without ambient noise. In other words, the presence of ambient noise significantly impacts word comprehension.

Regarding the effect of distance, when comparing the distances of 5M vs. 1M (5 m vs. 1 m), the Z value is −2.288 and the asymptotic significance is 0.022, which is below the *p*-value threshold of 0.05, allowing for the rejection of the null hypothesis. This suggests a significant difference in word recognition between the 5 m and 1 m distances. Practically, this indicates that the distance between the speaker and the listener significantly affects word comprehension.

Regarding the impact of the type of PPE used by the speaker on word recognition, in the relationships between PAPR vs. WM (PAPR vs. without mask), FFP2 vs. WM (FFP2 vs. without mask), and MasQX vs. WM (surgical mask vs. without mask), the Z values are −4.213, −4.216, and −4.152, respectively, all with an asymptotic significance of 0.000. As *p*-values are less than 0.05, we can reject the null hypothesis, indicating a statistically significant difference in word recognition when using any type of PPE compared to conditions without a mask. This implies that the use of PPE significantly affects word comprehension.

Thus, all the experimental conditions analyzed show significant differences in word recognition. Ambient noise, the distance between interlocutors, and the type of respiratory protective equipment (surgical mask, FFP2, PAPR) significantly influence participants’ ability to recognize spoken words.

## 4. Discussion

Based on the results from the 24 experimental scenarios, it was found that, whether combined or independently, all the variables analyzed (distance between interlocutors, presence of ambient noise, and use of respiratory PPE) influence the intelligibility of verbal messages.

The descriptive analysis of the data, which were grouped into intelligibility percentages, indicated a decrease in these percentages when combining ambient noise, distance, and PPE usage. The worst intelligibility percentages were observed in situations where the distance between interlocutors was 5 m or more, the speaker was wearing a PAPR, and the environment had noise levels equal to or greater than 45 dB(A). In this condition, 30% of the subjects recognized less than 20% of the words spoken, which equates to one out of five words spoken. This highlights the severity of such an impairment, where substantial portions of a message could be lost; in a clinical care setting, this could lead to serious communication issues and potentially fatal errors.

On the other hand, the data indicated that in environments with no noise (less than 25 dB(A)), at a distance of 1 m, and with the interlocutor not wearing a mask, recognition was always above 80%, with nearly 80% of participants correctly identifying 100% of the words spoken, and the remaining 20% identifying four out of every five words.

Both scenarios represent extremes that are rare in a clinical ICU environment, since in clinical situations such as pandemics or respiratory disease outbreaks, conditions where the interlocutor is not wearing a mask, is positioned less than one meter away, and is in a noisy environment are not plausible. Conversely, the occasional use of PAPR makes this condition plausible but uncommon.

Now, focusing on an analysis of the data from more likely scenarios in a real critical environment (ambient noise levels greater than 45 dB(A), the interlocutor wearing surgical or FFP2 masks, and a distance of two or more meters between interlocutors), the data showed that no listener recognized 100% of the words spoken under these conditions. Between 17% (surgical mask) and 35% (FFP2 mask) correctly recognized between 80 and 60% of the words, meaning they missed one or two out of five words spoken. In the condition with a distance of 5 m between interlocutors, most subjects recognized between 60% and 40% of the words with both types of masks, meaning most participants missed two to three words out of every five when at a distance of 5 m, in conditions of ambient noise above 45 dB(A), and when wearing any conventional mask (both respiratory and FFP2). This situation is frequent in a clinical environment, especially during emergencies, where the ambient noise level is higher than 45 dB(A), healthcare professionals wear surgical or FFP2 masks, and distances can exceed 5 m when healthcare professionals and patients are in different rooms or at opposite ends of the same room.

Overall, the Friedman and Wilcoxon tests confirm the individual impact each variable has on the number of correctly recognized words. Both tests show statistically significant relationships between the presence of ambient noise (45 dB(A) or more) and the recognition of spoken words, as well as between the type of PPE used and word recognition. However, there is a discrepancy between the Friedman and Wilcoxon tests regarding the relationship between distance and word comprehension. While it may seem that the results contradict each other, the two tests are applied differently. This discrepancy is plausible due to the treatment of the data; the Friedman test may have evaluated a more general analysis, using the mean of all distance values (1, 2, and 5 m) for the calculation. However, in the Wilcoxon test, two specific conditions (1 m vs. 5 m) were compared in greater detail, showing significant differences related to distance. Therefore, both tests can offer complementary, rather than mutually exclusive, perspectives. The Wilcoxon test confirms the observational data, pointing to a clear influence of distance on the correct interpretation of spoken words.

Among the three variables analyzed, ambient noise is perhaps the factor that most negatively influences the comprehension of verbal messages. Under low noise conditions (25 dB(A)), participants were able to recognize more than 80% of the words, regardless of distance or mask type. However, under clinical noise levels (50 dB(A) or more), words were only accurately recognized under specific conditions, such as close proximity combined with the use of surgical masks. It is the combination of these conditions with other dependent variables (distance and type of PPE used) that truly impacts word recognition. It is important to note that these scenarios closely resemble real clinical environments, where the effects of these factors are inseparable.

In this context, it is well known that clinical environments such as ED and ICU are among the noisiest [35,36,37]. In the ICU setting, several studies have aimed to measure noise levels and their effects on both patients and healthcare workers, reporting sound levels ranging from 45 dB(A) (the lowest recorded during the night) to 55 dB(A), with some studies indicating peaks exceeding 100 dB(A).

This noise is frequently generated by the healthcare activities themselves and is amplified by the number of people interacting in the same room. Studies like that of Yi et al. [38] indicate that, in the presence of noise, listeners performed worse when the speaker was wearing a surgical mask compared to when no mask was used. Similarly, Aliabadi et al. [28] highlight the influence of noisy environments on communication among healthcare professionals wearing face-covering masks.

Given the certainty of noise being present in emergency and intensive care units, the unique nature of the activities carried out in these settings, and the fact that our simulation lab tests reflect lower sound levels than the data shown in the literature, it is reasonable to assume that the conditions replicated in our simulated environments are indeed present in real clinical settings. In other words, it can be expected that verbal communication will be affected in noisy environments where healthcare workers are using face masks.

It can be inferred that distance causes a notable decrease in the intelligibility of verbal messages when background noise is present. At one meter, participants identified a high percentage of correct responses in the word recognition tests, maintaining their performance above the 80% threshold in most conditions. At two meters, intelligibility began to decline sharply, particularly under superficial noise conditions of 45 dB(A), where the use of respiratory protection devices, such as PAPR, significantly reduced the likelihood of participants correctly perceiving the message. At five meters, the problem became even more pronounced. In noisy conditions, 65% of participants failed to correctly understand more than 60% of the words spoken. In summary, both distance and background noise are factors that negatively impact the clarity of verbal messages, and as distance increases in noisy environments, the likelihood of the message being correctly understood diminishes, thereby compromising communication.

Evidence indicates that during the use of conventional PPE (face masks), it is recommended to reduce ambient noise to improve communication [39,40]. 

This idea of distance impacting the intelligibility of verbal messages has been widely studied and documented in the literature [41,42]. Studies such as this one apply the Inverse Square Law of Sound, which states that sound volume decreases as distance increases due to the dispersion of sound waves, which are transmitted through the air and reverberated by surrounding objects. Simply put, sound decreases by 6 dB(A) for every doubling of the distance from the sound source in an unobstructed environment. In practical terms, if someone speaks at 70 dB(A) at 1 m, the intensity will be approximately 64 dB(A) at 2 m and 58 dB(A) at 4 m. If the environment is noisy, echoes, or presents objects that act as barriers to sound waves, the loss can be even greater, affecting perception.

Like our results, various pieces of evidence show that the use of respiratory protective equipment significantly influences the accurate interpretation of words [43,44,45,46]. In our test, when the speaker was not wearing any type of respiratory protection, participants demonstrated near-perfect understanding under most conditions. However, although surgical masks and FFP2 masks allow for satisfactory word recognition, their performance decreases in the presence of noise and at greater distances, as expected, as the evidence has previously shown.

The evidence demonstrates that respiratory protective masks cause disturbances in the speech spectrum, which are reflected in qualities such as timbre and sound intensity. These disturbances manifest in sound attenuation and intelligibility tests, as well as in the perceived effort of the mask wearer [47,48,49]. This effect can often be attributed to the Lombard effect, which describes an instinctive reaction aimed at overcoming communication challenges [50]. According to this effect, in noisy environments, humans tend to increase the loudness of their voice, exerting more vocal effort to instinctively compensate for communication difficulties, as speakers attempt to maintain speech intelligibility. This effect arises unconsciously, and is often triggered when the speaker cannot hear their own voice or realizes they are not intelligible to the listener, prompting an automatic increase in vocal effort.

The use of PAPR poses a major problem, as it drastically reduces the ability of participants to understand spoken messages. In our study, the worst results regarding understanding the message delivered by the speaker were obtained when the speaker was wearing a PAPR; ratings in all conditions were worse, especially at distances greater than 2 m and in a noisy environment, where only 60% of the words were understood. At 5 m, understanding was less than 40% in a noiseless environment and less than 20% of words uttered in a noisy environment (>45dB(A)). These results are consistent with published evidence confirming an obvious loss of intelligibility among interlocutors when wearing a PAPR [51,52]. However, in our study, we quantified these negative effects.

These situations of impaired communication lead to an increase in cognitive load for healthcare workers and the adoption of compensatory behaviors, like the mentioned Lombard effect, mouth efforts, and gestural and visual support for oral communication [53]. The use of sign language or pictograms, together with technological support such as communication via headphones inside the PAPR caput, could help mitigate this negative effect on these teams.

Overall, our test shows that oral communication quality is negatively impacted by respiratory protective equipment, especially in noisy environments such as ICUs. The evidence suggests using devices that leave the face uncovered to compensate for the loss of verbal content due to the presence of a mask through enabling facial expressions to aid in communication [38]. However, while transparent masks allow for non-verbal cues by making facial expressions visible, they have poorer sound transmission than occlusive masks [54]. Nevertheless, the ability to see the speaker’s face compensates for this drawback, highlighting the importance of non-verbal communication in message transmission.

However, lip reading, which can be achieved with the use of clear face masks or through the use of PAPR, can be a communicative risk, rather than a reliable solution.

In situations where the intelligibility of the verbal message is low, visual cues from the speaker’s lips can sometimes lead to confusion. This is known as the McGurk effect [55], a perceptual phenomenon that highlights the interaction between hearing and vision in speech perception. It is an illusion in which the visual information received when watching someone speak alters the way a sound is perceived. If the auditory input is of poor quality but the visual input is clear, the listener is more likely to experience the McGurk effect. For example, when the auditory phoneme /ba/ is paired with the facial articulation of /ga/ (known as a viseme), it usually results in an illusory perception of ‘da’ [56]. In a noisy environment where verbal intelligibility is compromised by factors such as distance or the use of respiratory protective masks, the likelihood of misinterpretation due to the McGurk effect increases.

This effect can have serious consequences and is one explanation for the “look-alike, sound-alike” (LASA) phenomenon, which is a source of medication errors. LASA errors are exacerbated when the drugs involved are high-alert medications (opioids, insulin, anticoagulants, neuromuscular blocking agents, etc.). These errors occur when two medications have similar appearances, names, or pronunciations. Although the precise number of LASA errors is unknown, it is estimated that this mechanism could be involved in up to 25% of medication administration errors [57]. In particular, in 2008, the United States Pharmacopeia identified 3170 pairs of drug names that could potentially lead to LASA errors [58].

Therefore, enhancing communication among healthcare professionals in such environments, which are common in critical and emergency care settings, is a safety imperative.

Strategies to improve communication in the clinical setting should be implemented, such as using a microphone to amplify the voice of the speaker wearing PPE [59,60], especially when using PAPR. Visual aids, such as pictograms, written messages, and communication boards, should also be employed [61]. In addition, it is essential to enhance the communicative practices of healthcare professionals, such as establishing face-to-face communication, making eye contact, and using gestures in a non-distracting manner, such as pantomiming actions, providing positive or negative affirmations, or suggesting directions [62]. Clear messages should be conveyed, and confirmation of their receipt is crucial [63]. These strategies are low-cost, can be implemented in any scenario, and improve communication by reducing potential misunderstandings that may lead to patient safety or care issues.

## 5. Limitations

Despite the significance of the findings, our study presents certain limitations that should be considered. First, the quasi-experimental nature of the research, with a limited number of subjects, provides a preliminary analysis, but the results cannot be generalized to all users. It is also important to recognize the limitation arising from the selection of the number of participants based on previous studies, without a mathematical calculation.

No objective hearing tests were conducted on the participating subjects as part of the inclusion/exclusion criteria. They were only questioned regarding the presence or absence of hearing pathologies.

Another limitation to acknowledge is related to the environment in which the study was conducted, as it was a simulated setting rather than a real context. Although clinical conditions were largely reproduced, they may not fully reflect the reality of intensive care units. Furthermore, the combined interaction between the dependent variables (noise, distance, and PPE device) prevents a precise determination of the individual influence of each on the independent variable. However, in real-world settings, this relationship is natural and difficult to isolate.

These methodological limitations suggest that, while the study provides valuable insights into the factors affecting intelligibility in high-risk environments, further investigation is needed into the relationship between verbal and non-verbal communication and the impact of real clinical environments, and a larger, more diverse sample is needed to increase the robustness of the findings. Additionally, it would be beneficial to explore more technological strategies to improve communication and assess the effect of these findings in critical situations, where effective communication is vital for patient safety.

## 6. Conclusions

In conclusion, this study provided significant findings regarding the factors affecting intelligibility in high-risk clinical environments, particularly in intensive care units, where the use of personal protective equipment (PPE) and ambient noise are prevalent. The results support the hypothesis that the combination of noise, distance, and the type of PPE has a significant impact on speech clarity and understanding among healthcare professionals. It was observed that the use of protective devices, such as Powered Air-Purifying Respirators (PAPR), leads to a notable decrease in intelligibility. When combined with high noise levels (>45 dB(A)) and a distance of more than 2 m between interlocutors, intelligibility was reduced by over 40%.

This study also identified the importance of both visual and verbal communication strategies, such as the use of visual aids, gestures, and eye contact, to mitigate the negative effects of communication barriers. Although the analysis was conducted in a simulated environment, the findings highlight the relevance of these factors in real-life situations, suggesting that in actual clinical contexts, these communication challenges may compromise patient safety. However, further research is needed to more deeply explore the interactions between verbal and non-verbal communication and to assess how different technological strategies can optimize communication in these critical environments.

## Figures and Tables

**Table 1:** Demographic characteristics of the subjects.

Gender		Age					
		Mean	Median	Mode	ST	Max	Min
Women	16 (69.6%)	24.9	21.5	21	7.92	47	21
Men	7 (30.4%)	26.1	24	22	6.04	37	21

**Table 2:** Mean difference. Condition 0–Condition 1.

1 m				2 m				5 m				MEAN
WM	SM	FFP2	PAPR	WM	SM	FFP2	PAPR	WM	SM	FFP2	PAPR	
0.65	0.65	1.38	1.34	1.34	1.17	1.32	0.66	1.13	1.63	1.69	1.18	1.18

WM: without a mask; SM: surgical mask; PAPR: Positive Pressure Air Purifiers.

**Table 3:** Frequency table of percentage of success by distance.

Distance: 1M								
	WM		SM		FFP2		PAPR	
% Intell.	Without Noise	With Noise	Without Noise	With Noise	Without Noise	With Noise	Without Noise	With Noise
**Excellent**	**18 (79.3%)**	7 (30.4%)	**15 (65.2%)**	4 (17.3%)	**16 (69.6%)**	0	8 (34.8%)	0
**Good**	5 (21.7%)	**12 (52.2%)**	8 (34.8%)	**15 (65.2%)**	7 (30.4%)	11 (47.8%)	**12 (52.2%)**	4 (17.4%)
**Regular**	0	4 (17.4%)	0	4 (17.4%)	0	**8 (34.8%)**	3 (13%)	**12 (52.2%)**
**Poor**	0	0	0	0	0	4 (17.4%)	0	7 (30.4%)
**Bad**	0	0	0	0	0	0	0	
**Very Bad**	0	0	0	0	0	0	0	0
**Distance: 2M**								
**% Intell.**	**Without Noise**	**With Noise**	**Without Noise**	**With Noise**	**Without Noise**	**With Noise**	**Without Noise**	**With Noise**
**Excellent**	**14 (60.8%)**	2 (8.7%)	11 (47.8%)	0	7 (30.4%)	0	0	0
**Good**	9 (39.2%)	**13 (56.5%)**	**12 (52.2%)**	**11 (47.8%)**	**11(47.8%)**	3 (13%)	**11 (47.8%)**	4 (17.4%)
**Regular**	0	4 (17.4%)	0	8 (34.8%)	5 (21.7%)	**12 (52.2%)**	6 (26.1%)	**8 (34.8%)**
**Poor**	0	4 (17.4%)	0	4 (17.4%)	0	8 (34.8%)	6 (26.1%)	8 (34.8%)
**Bad**	0	0	0	0	0	0	0	3 (13%)
**Very Bad**	0	0	0	0	0	0	0	0
**Distance: 5M**								
**% Intell.**	**Without Noise**	**With Noise**	**Without Noise**	**With Noise**	**Without Noise**	**With Noise**	**Without Noise**	**With Noise**
**Excellent**	**15 (66.2%)**	4 (17.4%)	3 (13%)	0	5 (21.7%)	0	0	0
**Good**	4 (17.4%)	4 (17.4%)	**16 (69.5%)**	3 (13%)	**10 (43.5%)**	0	0	0
**Regular**	4 (17.4%)	**11 (47.8%)**	4 (17.4%)	4 (17.4%)	8 (34.8%)	8 (34.8%)	**12 (52.2%)**	0
**Poor**	0	4 (17.4%)	0	**12 (52.2%)**	0	**11 (47.8%)**	8 (34.8%)	**12 (52.2%)**
**Bad**	0	0	0	4 (17.4%)	0	4 (17.4%)	3 (13%)	4 (17.4%)
**Very Bad**	0	0	0	0	0	0	0	7 (30.4%)

% Intell (intelligibility percentage): Excellent (100–91%); Good (90–81%); Regular (80–61%); Poor (60–41%); Bad (40–21%) Very Bad (<21%). Shaded: the highest value is marked. Without noise: only ambient noise. Noise level <25 dB(A). With noise: added ambient ICU noise. Noise level > 45 dB(A).

**Table 4:** Frequency table of percentage of success at each distance protective equipment was used by the emitter.

No Mask						
	1M		2M		5M	
% Intell.	Without Noise	With Noise	Without Noise	With Noise	Without Noise	With Noise
**Excellent**	**18 (78.3%)**	7 (30.4%)	**14 (60.9%)**	2 (8.7%)	**15 (65.2%)**	4 (17.4%)
**Good**	5 (21.7%)	**12 (52.2%)**	9 (39.1%)	**13 (56.5%)**	4 (17.4%)	4 (17.4%)
**Regular**	0	4 (17.4%)	0	4 (17.4%)	4 (17.4%)	**11 (37.8%)**
**Poor**	0	0	0	4 (17.4%)	0	4 (17.4%)
**Bad**	0	0	0	0	0	0
**Very Bad**	0	0	0	0	0	0
**Surgical Mask.**						
**% Intell.**	**Without Noise**	**With Noise**	**Without Noise**	**With Noise**	**Without Noise**	**With Noise**
**Excellent**	**15 (65.2%)**	4 (17.4%)	11 (47.8%)	0	3 (13%)	0
**Good**	8 (34.8%)	**15 (65.2%)**	**12 (52.2%)**	**11 (47.8%)**	**16 (69.5%)**	3 (13%)
**Regular**	0	4 (17.4%)	0	8 (34.8%)	4 (17.4%)	4 (17.4%)
**Poor**	0	0	0	4 (17.4%)	0	**12 52.2%)**
**Bad**	0	0	0	0	0	4 (17.4%)
**Very Bad**	0	0	0	0	0	0
**FFP2**						
**% Intell.**	**Without Noise**	**With Noise**	**Without Noise**	**With Noise**	**Without Noise**	**With Noise**
**Excellent**	**16 (69.6%)**	0	7 (30.4%)	0	5 (21.7%)	0
**Good**	7 (30.4%)	**11 (48.8%)**	**11 (48.8%)**	3 (13%)	**10 (43.5%)**	0
**Regular**	0	8 (34.7%)	5 (21.7%)	**12 (52.2%)**	8 (34.7%)	8 (34.7%)
**Poor**	0	4 (17.4%)	0	8 (34.7%)	0	**11 (48.8%)**
**Bad**	0	0	0	0	0	4 (17.4%)
**Very Bad**	0	0	0	0	0	0
**PAPR.**						
**% Intell.**	**Without Noise**	**With Noise**	**Without Noise**	**With Noise**	**Without Noise**	**With Noise**
**Excellent**	8 (34.8%)	0	0	0	0	0
**Good**	**12 (52.2%)**	4 (17.4%)	**11 (47.8%)**	4 (17.4%)	0	0
**Regular**	3 (13%)	**12 (52.2%)**	6 (26.1%)	**8 (34.8%)**	**12 (52.2%)**	0
**Poor**	0	7 (30.4%)	6 (26.1%)	8 (34.8%)	8 (34.8%)	**12 (52.2%)**
**Bad**	0	0	0	3 (13%)	3 (13%)	4 (17.4%)
**Very Bad**	0	0	0	0	0	7 (30.4%)

% Intell (intelligibility percentage): Excellent (100–91%); Good (90–81%); Regular (80–61%); Poor (60–41%); Bad (40–21%); Very Bad (<21%). Shaded: the highest value is marked. Without noise: only ambient noise. Noise level <25 dB(A). With noise: added ambient ICU noise. Noise level > 45 dB(A).

**Table 5:** Frequency table showing the percentage of success under ambient noise conditions.

Without Ambient Sound Added; Noise Level <25 dB(A)												
	1M				2M				5M			
% Intell.	WM	SM	FFP2	PAPR	WM	SM	FFP2	PAPR	WM	SM	FFP2	PAPR
**Excellent**	**18 (78.3%)**	**15 (65.2%)**	**16 (69.5%)**	8 (34.8%)	**14 (60.9%)**	11 (47.8%)	7 (30.4%)	**0**	**15 (65.2%)**	3 (13%)	5 (21.7%)	0
**Good**	5 (21.7%)	8 (34.8%)	7 (30.4%)	**12 (52.2%)**	9 (39.1%)	**12** **(52.2%)**	**11 (47.8%)**	**11** **(47.8%)**	4 (17.4%)	**16** **(69.5%)**	**10** **(43.5%)**	0
**Regular**	0	0	0	3 (13%)	0	0	5 (21.7%)	**6** **(26.1%)**	4 (17.4%)	4 (17.4%)	8 (34.8%)	**12** **(52.2%)**
**Poor**	0	0	0	0	0	0	0	6 (26.1%)	0	0	**0**	8 (34.8%)
**Bad**	0	0	0	0	0	0	0	0	0	0	0	3 (13%)
**Very Bad**	0	0	0	0	0	0	0	0	0	0	0	0
**With ambient sound added; Noise level < 45 dB(A)**												
**% Intell.**	**WM**	**SM**	**FFP2**	**PAPR**	**WM**	**SM**	**FFP2**	**PAPR**	**WM**	**SM**	**FFP2**	**PAPR**
**Excellent**	7 (30.4%)	4 (17.4%)	0	0	2 (8.7%)	0	0	0	4 (17.4%)	0	0	0
**Good**	**12** **(52.2%)**	**15** **(65.2%)**	**11** **(47.8%)**	4 (17.4%)	**13** **(56.5%)**	**11** **(47.8%)**	3 (13%)	4 (17.4%)	4 (17.4%)	3 (13%)	0	0
**Regular**	4 (17.4%)	4 (17.4%)	8 (34.8%)	**12** **(52.2%)**	4 (17.4%)	8 (34.8%)	**12** **(52.2%)**	**8** **(34.8%)**	**11** **(47.8%)**	4 (17.4%)	8 (34.8%)	**0**
**Poor**			4 (17.4%)	7 (30.%)	4 (17.4%)	4 (17.4%)	8 (34.8%)	8 (34.8%)	4 (17.4%)	**12** **(52.2%)**	**11** **(47.8%)**	**12** **(52.2%)**
**Bad**								3 (13%)		4 (17.4%)	4 (17.4%)	4 (17.4%)
**Very Bad**												7 (30.4%)

% Intell (intelligibility percentage): Excellent (100–91%); Good (90–81%); Regular (80–61%); Poor (60–41%); Bad (40–21%); Very Bad (<21%). Shaded: the highest value is marked. Without noise: only ambient noise. Noise level <25 dB(A). With noise: added ambient ICU noise. Noise level >45 dB(A).

## Data Availability

The original contributions presented in the study are included in the article/Supplementary Materials; further inquiries can be directed to the corresponding author.

## Supplementary Materials

The following supporting information can be downloaded at: https://www.mdpi.com/article/10.3390/healthcare13040398/s1, Table S1: Selected phonemically balanced word sequences; Table S2: Descriptive Statistics of Correct Responses; Table S3: Friedman Test; Table S4: Wilcoxon Test; Figure S1: Study Protocol Diagram.
