# Supplementary material for: Effects of Distance, Noise, and Personal Respiratory Protective Equipment on Speech Comprehension in Simulated Critical Care Setting
Source: Healthcare (Basel). 2025 Feb 12;13(4):398. doi: 10.3390/healthcare13040398 (PMC11855685; doi:10.3390/healthcare13040398)
Supplement: Supplementary file 1 [file healthcare-13-00398-s001.zip › healthcare-3394677-supplementary.pdf]

Table. S1 Selected phonemically balanced word sequences.

A-1	A-2	A-3	B-1	B-2	B-3	C-1	C-2	C-3	D-1	D-2	D-3
Libro	Mano	Beso	Pollo	Valija	Percha	Fantasma	Cuna	Goma	Vino	Oso	Pala
Mano	Gallina	Moto	Pipa	Nena	Cubos	Lata	Goma	Buzo	Pila	Brazo	Barco
Pan	Pan	Luna	Pileta	Pelo	Uvas	Chino	Castillo	Cuna	Pared	Trenes	Pulpo
Lampara	Bandera	mano	Queso	Paleta	Playa	Hoja	Tele	Hoja	Hueso	Pluma	Cadena
Bota	Torta	bota	Sopa	Pollo	Pecera	Princesa	Pizza	Ballena	Trenza	Sirena	Sangre

Table. S2. Descriptive Statistics of Correct Responses.

Condition 0 No Added Ambient Noise.

	1 meter				2 meters				5 meters			
	**WM**	**SM**	**FFP2**	**PAPR**	**WM**	**SM**	**FFP2**	**PAPR**	**WM**	**SM**	**FFP2**	**PAPR**
**Mean**	4,78	4,65	4,69	4,21	4,6	4,47	4,1	3,22	4,48	3,9	3,86	2,39
**Median**	5	5	5	4	5	4	4	3	5	4	4	3
**Mode**	5	5	5	4	5	4	4	4	5	4	4	3
**ST**	0,42	0,49	0,47	0,67	0,49	0,51	0,73	0,85	0,79	0,56	0,76	0,72
**Skewness**	-1,46	-0,68	-0,91	-0,28	1E-06	2E-06	0,001	9E-05	4E-06	4E-05	0,001	7E-05
**Kurtosis**	0,16	-1,68	-1,29	-0,62	-1,8	-1,99	-1,1	-1,42	-0,51	0,28	-1,15	-0,71
**Min**	4	4	4	3	4	4	3	2	3	3	3	1
**Max**	5	5	5	5	5	5	5	5	5	5	5	3
**Shapiro-Willk test (p>0.05)**												
**W-stat**	0,5227	0,61268	0,59043	0,79295	0,622	0,639	0,812	0,760	0,661	0,735	0,809	0,753
**p-value**	2 E-07	1,8E-06	1,05E-6	0,00039	0,000	0,000	0,001	0,000	0,000	0,000	0,001	0,000
**Normality**	No	No	No	No	No	No	No	No	No	No	No	No

Condition 1. Ambient Noise Added.

	1 meter				2 meters				5 meters			
	**WM**	**SM**	**FFP2**	**PAPR**	**WM**	**SM**	**FFP2**	**PAPR**	**WM**	**SM**	**FFP2**	**PAPR**
**Mean**	4,13	4	3,31	2,87	3,56	3,3	2,78	2,56	3,35	2,27	2,17	1,21
**Median**	4	4	3	3	4	3	3	3	3	2	2	2
**Mode**	4	4	4	3	4	4	3	2	3	2	2	2
**ST**	0,69	0,6031	0,76	0,69	0,896	0,76	0,67	0,94	0,982	0,9153	0,717	0,9023
**Skewness**	0,0045	0,00011	0,00014	0,00044	0,006	0,001	0,003	0,014	0,029	0,002	0,001	0,0003
**Kurtosis**	-0,84	-0,12	-1,025	-0,8464	-0,53	-1,02	-0,75	-0,85	-0,76	-0,37	-0,96	-1,57
**Min**	3	3	2	2	2	2	2	1	2	1	1	0
**Max**	5	5	4	4	5	4	4	4	5	4	3	2
**Shapiro-Willk test (p>0.05)**												
**W-stat**	0,8041	0,7656	0,7723	0,8041	0,8108	0,772	0,791	0,887	0,852	0,844	0,803	0,722
**p-value**	0,0004	0,0001	0,0001	0,0004	0,001	0,001	0,001	0,014	0,003	0,002	0,001	2E-05
**Normality**	No	No	No	No	No	No	No	No	No	No	No	No

WM: Without a mask; SM: surgical mask; PAPR: Positive Pressure Air Purifiers

Table S3. Friedman Test.				
**Respiratory Protective Equipment Used by Emitter vs Recognized Words**				
	**WM**	**SM**	**FFP2**	**PAPR**
**Average Ratio**	3,98	2,98	2,04	1
**Chi-Square**	68,128			
**p-value**	0,000001			
**Sound Environmental Conditions vs Recognized Words**				
	**Without Noise (<25 dB(A))**		**With Noise (>45 dB(A))**	
**Average Ratio**	2		1	
**Chi-Square**	23			
**p-value**	0,000001			
**Sender-Receiver Distance vs Recognized Words**				
	1 meter	2 meters	3 meters	
**Average Ratio**	2,26	2,02	1,72	
**Chi-Square**	3,528			
**p-value**	0,171			

Table S4. Wilcoxon Test.					
**Wilcoxon Test**					
	**Without-Noise** vs With-Noise	Distance 5M vs 1M	PAPR vs **WM**	FFP2 vs **WM**	MasQX vs **WM**
**Z**	-4,202	-2,288	-4,213	-4,216	-4,152
**Asymptotic Significance (bilateral)**	0,0000	0,022	0,000	0,000	0,000

Figure 1.

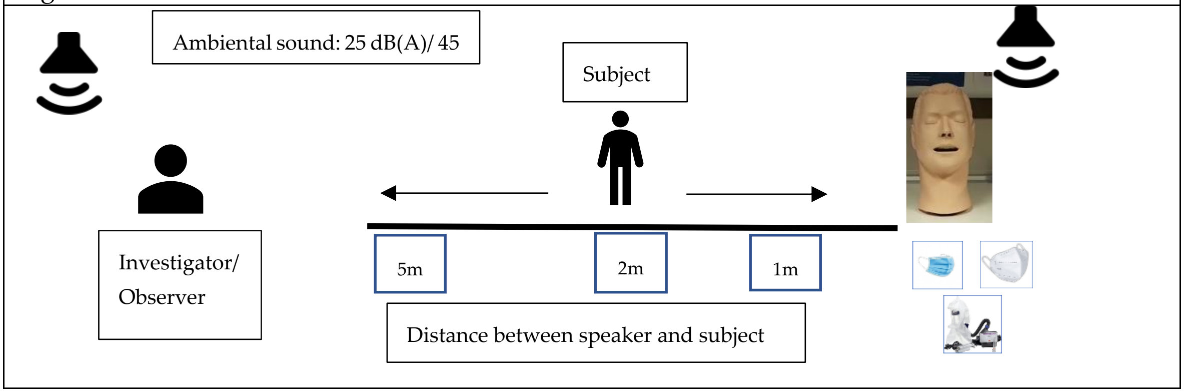

Ambiental sound: 25 dB(A)/ 45
Subject
Investigator/
Observer
5m
2m
1m
Distance between speaker and subject
